# Human Embryonic Mesenchymal Stem Cell-Derived Conditioned Medium Rescues Kidney Function in Rats with Established Chronic Kidney Disease

**DOI:** 10.1371/journal.pone.0038746

**Published:** 2012-06-19

**Authors:** Arianne van Koppen, Jaap A. Joles, Bas W. M. van Balkom, Sai Kiang Lim, Dominique de Kleijn, Rachel H. Giles, Marianne C. Verhaar

**Affiliations:** 1 Department of Nephrology and Hypertension, University Medical Center Utrecht, Utrecht, the Netherlands; 2 Institute of Medical Biology, A*STAR, Singapore, Republic of Singapore; 3 Department of Experimental Cardiology, University Medical Center Utrecht, Utrecht, the Netherlands; INSERM, France

## Abstract

Chronic kidney disease (CKD) is a major health care problem, affecting more than 35% of the elderly population worldwide. New interventions to slow or prevent disease progression are urgently needed. Beneficial effects of mesenchymal stem cells (MSC) have been described, however it is unclear whether the MSCs themselves or their secretome is required. We hypothesized that MSC-derived conditioned medium (CM) reduces progression of CKD and studied functional and structural effects in a rat model of established CKD.

CKD was induced by 5/6 nephrectomy (SNX) combined with L-NNA and 6% NaCl diet in Lewis rats. Six weeks after SNX, CKD rats received either 50 µg CM or 50 µg non-CM (NCM) twice daily intravenously for four consecutive days. Six weeks after treatment CM administration was functionally effective: glomerular filtration rate (inulin clearance) and effective renal plasma flow (PAH clearance) were significantly higher in CM vs. NCM-treatment. Systolic blood pressure was lower in CM compared to NCM. Proteinuria tended to be lower after CM. Tubular and glomerular damage were reduced and more glomerular endothelial cells were found after CM. DNA damage repair was increased after CM. MSC-CM derived exosomes, tested in the same experimental setting, showed no protective effect on the kidney. In a rat model of established CKD, we demonstrated that administration of MSC-CM has a long-lasting therapeutic rescue function shown by decreased progression of CKD and reduced hypertension and glomerular injury.

## Introduction

The number of patients with chronic kidney disease (CKD) is rising to epidemic proportions [1]. In 2008, the median prevalence of CKD was 7% in persons aged 30 years or older. In persons aged 64 years or older prevalence of CKD varied from 23% to 36% and is still increasing [2]. The ensuing end-stage kidney disease, as well as the associated increase in cardiovascular risk, has significant socio-economic and major public health implications [3]. Nowadays, renal replacement therapy consists of either dialysis or, preferably, kidney transplantation, which is severely limited due to donor shortage. Both renal replacement strategies are associated with increased morbidity and mortality [4]. Consequently, new interventions to slow or prevent CKD progression are being actively pursued. Mesenchymal stem cell (MSC)-based therapies have been proposed as potential new treatment modality.

Administration of MSCs has been shown to offer protection in several models of acute kidney injury [5]. Some data demonstrate a positive effect of MSC treatment on the loss of renal function in early stage CKD models as well [6]. In these studies, however, incorporation and trans-differentiation of injected MSCs were rare events, suggesting that MSCs primarily have a supportive function, probably by secreting growth factors and cytokines [7]. Such a paracrine mode of action has the therapeutic potential for cell-free treatment strategies using MSC-secreted factors. Importantly, if administration of MSC-derived secreted factors can reduce CKD progression, this may have major clinical relevance as such therapy could overcome problems associated with (allogenic) MSC administration such as immune incompatibility, MSC maldifferentiation [8], [9] and tumorgenicity [10], [11]. Thus far, the *in vivo* effects of MSC-secreted factors have only been studied in acute kidney disease. Bi et al. demonstrated that administration of conditioned medium (CM) from bone marrow-derived MSCs in a model of acute kidney injury (AKI) increased survival and limited renal injury, assessed as decreased blood urea nitrogen (BUN) concentrations [12]. Geishara et al., however, could not confirm such beneficial effects of MSC-CM in experimental AKI [13].

The relevance of these observations in AKI to CKD is unclear. To our knowledge, the effect of MSC secreted factors has not been investigated in a model of established CKD.

The paracrine factors secreted by MSC that are responsible for the (reno) protective effects have not been fully elucidated. Next to immunomodulatory and anti-inflammatory properties of MSC, important roles were suggested for proangiogenic factors like vascular endothelial growth factor (VEGF), hepatocyte growth factor (HGF) and insulin-like growth factor (IGF) [14]–[17]. Recent reports support a central role for microvesicles [18], [19] or exosomes [20] in MSC-mediated tissue repair. In experimental myocardial infarction the cardio-protective effects of human embryonic MSC-CM were attributed to exosomes [21], [22].

We hypothesized that MSC-secreted factors have a therapeutic rescue function by supporting renal repair and hence renal function and thus reduce progression of established CKD. Therefore, in the setting of established CKD induced by subtotal nephrectomy, the effects of repeated intravenous delivery of human embryonic MSC-derived CM on renal hemodynamics and injury were studied.

## Results

### CM Treatment Reduces CKD Progression

**Figure 1:**
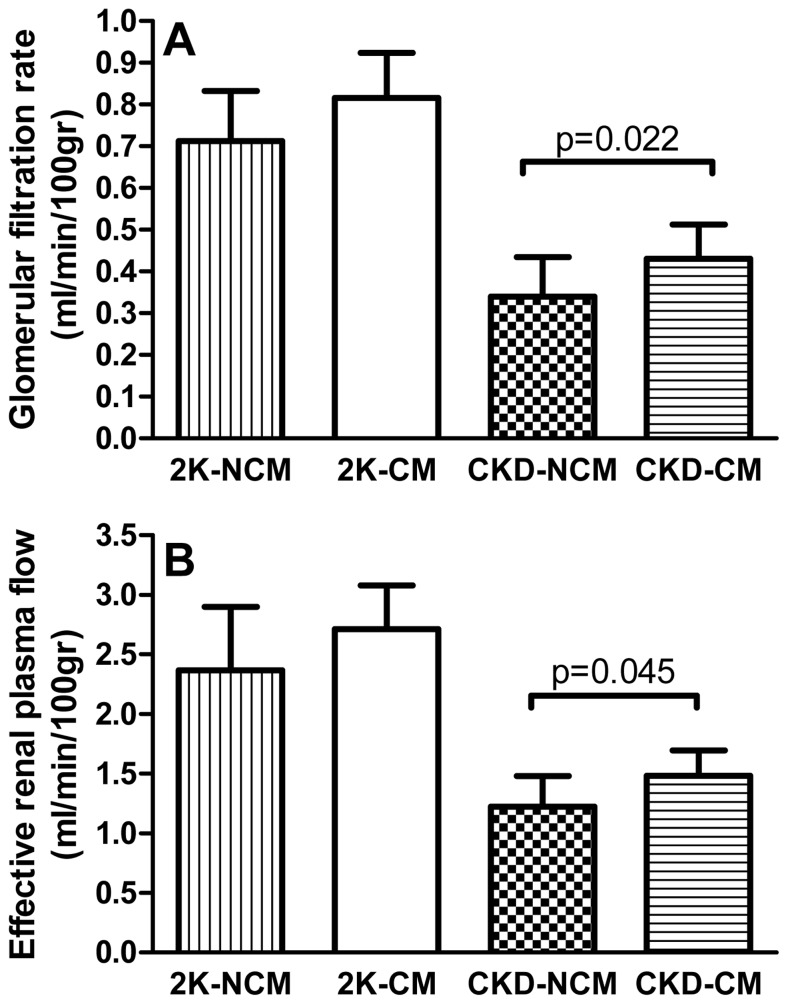
CM treatment increases kidney function. CM treatment increased glomerular filtration rate (GFR) (A) and effective renal plasma flow (ERPF) (B) in CKD. 2K-NCM (n = 6); 2K-CM (n = 6); CKD-CM (n = 13); CKD-NCM (n = 13). Post-hoc test p-value is shown.

Six weeks after SNX, CKD was established and rats received treatment with CM or NCM. At 6 weeks after treatment (12 weeks after SNX), treatment with CM resulted in significantly higher GFR and ERPF compared to treatment with NCM (both *p*<0.05) at t = 12 weeks (figure 1). No differences were observed in hematocrit, mean arterial pressure (MAP), renal vascular resistance (RVR), filtration fraction (FF) and fractional excretion of sodium and potassium between CM and NCM (table 1). We observed no significant differences in GFR and ERPF in healthy controls between CM and NCM treatment. Exosome treatment had no effect on CKD progression (table S1).

**Table 1:** Terminal kidney function measurements.

	2K-NCM n = 6	2K-CM n = 6	CKD-NCM n = 13	CKD-CM n = 13
Body weight (g)	442±43	432±23	364±22*	355±16*
MAP (mm Hg)	99±9	93±13	122±22*	135±19*
RBF (ml/min/100 g)	4.64±1.37	5.22±0.66	2.10±0.49*	2.56±0.43*
RVR (mm Hg/ml/min)	4.9±1.1	4.1±0.6	17±9*	15±4*
Hematocrit	50±1	49±2	45±2*	46±2*
FF	0.32±0.03	0.30±0.05	0.28±0.05	0.29±0.05
FeNa (%)	0.44±0.24	0.38±0.24	1.59±1.79*	1.55±0.77*
FeK (%)	13±6	14±6	38±11*	45±13*

Mean±SD *P<0.05: 2K vs. CKD.

MAP = mean arterial pressure. RBF = renal blood flow. RVR = renal vascular resistance. FF = filtration fraction. FeNa = fractional excretion of sodium. FeK = fractional excretion of potassium.

### CM Treatment Reduces the Increase of Systolic Blood Pressure (SBP) in CKD Rats

**Figure 2:**
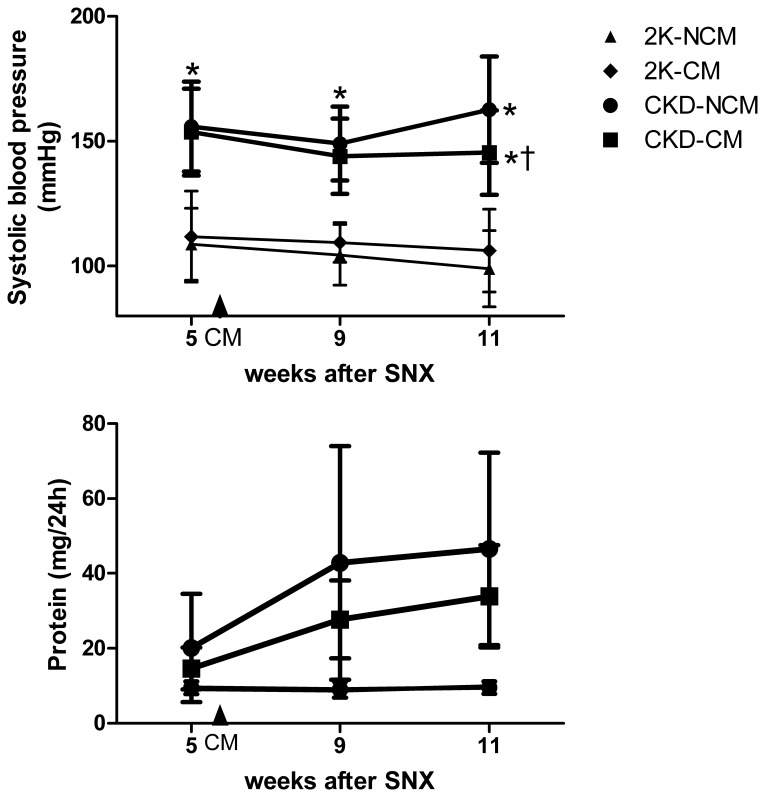
CM treatment decreased systolic blood pressure (A) and proteinuria (B) in CKD. 2K-NCM (n = 6); ♦ 2K-CM (n = 6); ▪ CKD-CM (n = 13); • CKD-NCM (n = 13). *P<0.05: CKD vs. 2K, † P<0.05: CKD-CM vs. CKD-NCM.

CKD animals showed significant hypertension compared to healthy controls (figure 2a). SBP was significantly lower in CKD-CM treated rats compared to CKD-NCM treated rats at week 11 (146±17 vs. 163±21 mm Hg; *p*<0.05, figure 2a). CKD rats showed more proteinuria compared to healthy controls. Protein excretion tended to be lower in CKD-CM-treated compared to CKD-NCM-treated rats (figure 2b; *p* = 0.071). No differences were observed in urea and creatinine clearance between CKD-CM and CKD-NCM rats at wk 11 (table 2). Plasma creatinine was increased in CKD-NCM compared to CKD-CM (*p* = 0.05). Importantly, CM or NCM administration did not influence SBP, proteinuria or creatinine clearance in healthy control rats. Exosome treatment had no effect of SBP (table S2).

**Table 2:** Longitudinal measurements after CM treatment at week 6 after SNX.

	2K-NCM n = 6	2K-CM n = 6	CKD-NCM n = 13	CKD-CM n = 13	P (CKD-CM vs. CKD-NCM)
**Urea (mmol/L)**					
wk 5	5.7±0.9	6.2±1.0	10.3±2.4*	10.2±2.5*	0.854
wk 9	5.9±0.5	5.9±1.1	10.8±2.2*	12.2±1.9*	0.058
wk 11	6.5±0.8	6.9±1.5	9.7±1.9*	9.9±1.8*	0.701
**Plasma creatinine (µmol/L)**					
wk 5	20.1±3.3	20.8±1.1	49.7±8.8*	48.6±9.9*	0.948
wk9	21.8±6.6	25.6±4.5	44.9±11.4*	42.9±6.6*	0.558
wk11	28.9±7.5	22.7±3.1	51.0±14.2*	42.8±6.9*	0.050
**Creatinine clearance (ml/min)**					
wk 5	4.18±0.52	4.13±1.31	1.18±0.22*	1.13±0.25*	0.795
wk 9	3.84±1.27	3.00±0.44*	1.48±0.36*	1.43±0.21*	0.801
wk 11	2.84±0.74	3.27±0.93	1.32±0.35*	1.50±0.28*	0.386

Week numbers indicate the week after SNX. Week 5 represents the week before treatment.

* P<0.05 vs. respective 2K controls. Posthoc p-value is shown.

### CM Increases the Number of Glomerular Endothelial Cells and Reduces Glomerulosclerosis and Tubular Damage in CKD Rats

**Figure 3:**
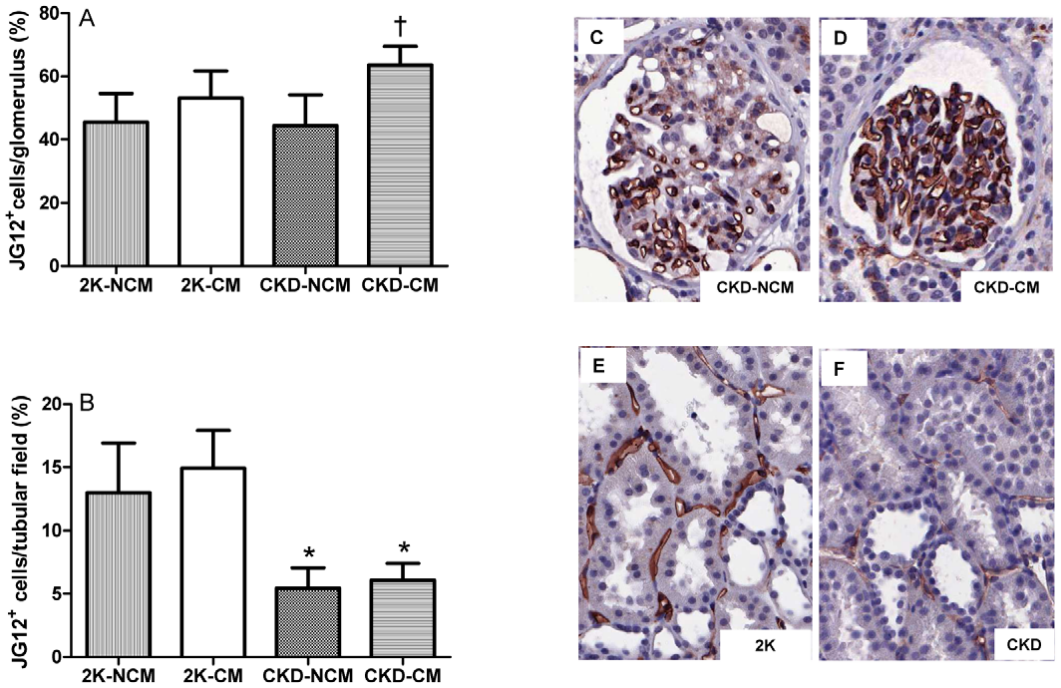
CM treatment increased the number of glomerular endothelial cells (A, C-D), but did not increase tubular endothelial cell number (B, E-F) shown by a JG12 staining. 2K-NCM (n = 6); 2K-CM (n = 6); CKD-NCM (n = 13); CKD-CM (n = 13). *P<0.05: CKD vs. 2K, †P<0.05: CKD-CM vs. CKD-NCM.

**Figure 4:**
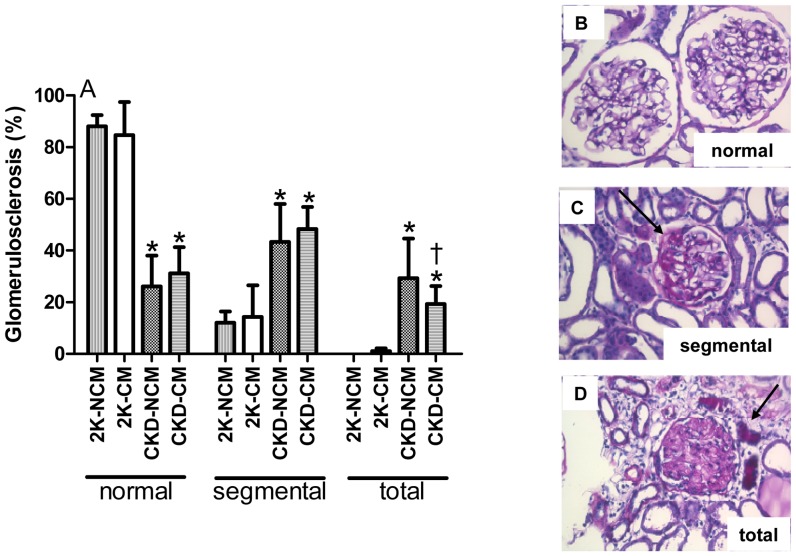
Glomerulosclerosis was reduced by CM treatment. On PAS-stained sections, glomeurosclerosis was reduced after CM treatment (A). Normal (B), segmental (C) and totally sclerotic are shown (D). Black arrows indicate sclerotic areas. 2K-NCM (n = 6); 2K-CM (n = 6); CKD-NCM (n = 13); CKD-CM (n = 13). *P<0.05: CKD vs. 2K, †P<0.05: CKD-CM vs. CKD-NCM.

**Figure 5:**
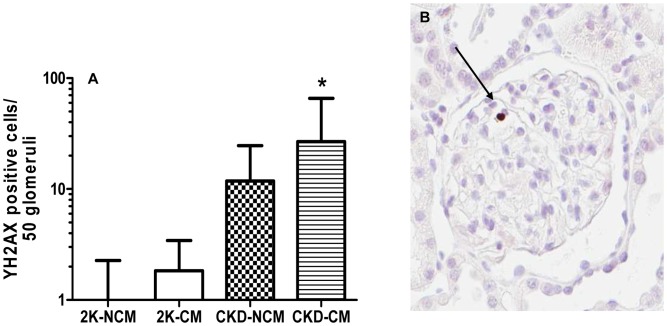
CM treatment increased DNA damage repair. After logarithmic transformation, more γ-H2AX positive cells, indicating glomerular nuclei undergoing DNA damage repair, were found after CM treatment in CKD (A). Black arrow indicates positive nucleus (B). 2K-NCM (n = 6); 2K-CM (n = 6); CKD-NCM (n = 13); CKD-CM (n = 13). *P<0.05: CKD vs. 2K.

The percentage of glomerular endothelial cells, (JG12 positive), was significantly higher in CKD-CM compared to CKD-NCM- treated rats (*p*<0.05, fig. 3). Both CKD-CM and CKD-NCM-treated rats showed marked glomerulosclerosis as compared to healthy rats with respectively 31% and 26% normal, non-sclerotic glomeruli as compared to more than 85% normal glomeruli in healthy controls. Comparing the number of partly and totally sclerotic glomeruli between the CKD groups reveals a favourable shift with significantly more partly sclerotic glomeruli and less totally sclerotic glomeruli in CKD-CM compared to CKD-NCM-treated rats (figure 4). DNA damage and repair was measured by induction of cellular γ-H2AX; γ-H2AX expression has been established as a sensitive indicator of clonogenic survival after tissue damage and induction of DNA damage repair [23], [24]. We noted with interest that CM significantly increased glomerular γ-H2AX induction in CKD rats compared to healthy rats whereas CKD-NCM rats were not different compared to healthy rats (figure 5). The numbers of glomerular proliferating cells as determined by Ki67 and glomerular inflammatory CD3^+^ and ED-1^+^ cells were not different between the CKD-CM and CKD-NCM -treated rats (table 3).

**Table 3:** Renal morphology.

	2K-NCM n = 6	2K-CM n = 6	CKD-NCM n = 13	CKD-CM n = 13
**Glomerular measurements**				
**Ki 67^+^ cells/glomerulus**	3.85±1.01	4.17±1.38	5.11±1.52	5.99±1.60*
**ED1^+^ cells/glomerulus**	1.33±0.51	1.33±0.51	2.70±1.34*	2.59±0.84*
**CD3^+^ cells/glomerulus**	0.14±0.08	0.09±0.07	0.46±0.36*	0.34±0.13
**Tubular measurements**				
**ED1^+^ cells/tubular field**	12.7±0.8	13.5±3.5	32.9±10.2*	34.3±3.9*
**CD3^+^ cells/20 tubular fields**	2.2±0.9	1.4±0.7	49.3±23.7*	48.8±8.0*
**TUNEL^+^cells/tubular field**	0.65±0.68	0.23±0.14	1.75±1.12	1.30±0.77*

**Figure 6:**
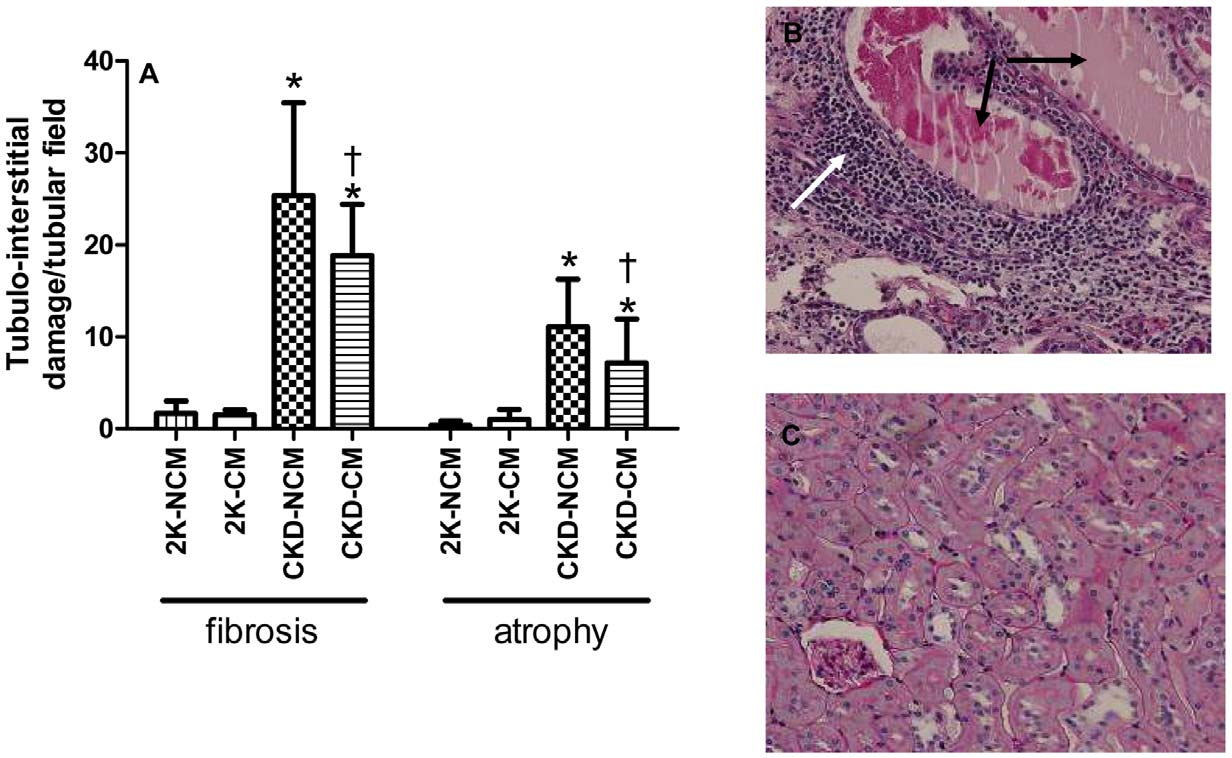
Tubulo-interstitial damage on PAS-stained sections was reduced after CM treatment in CKD rats. CM treatment decreased tubular fibrosis and atrophy (A). Differences between CKD (B) and healthy (C) tubular kidney tissue are shown. White arrow indicates infitration, black arrows showing proteincasts. 2K-NCM (n = 6); 2K-CM (n = 6); CKD-NCM (n = 13); CKD-CM (n = 13). *P<0.05: CKD vs. 2K, †P<0.05: CKD-CM vs. CKD-NCM.

**Figure 7:**
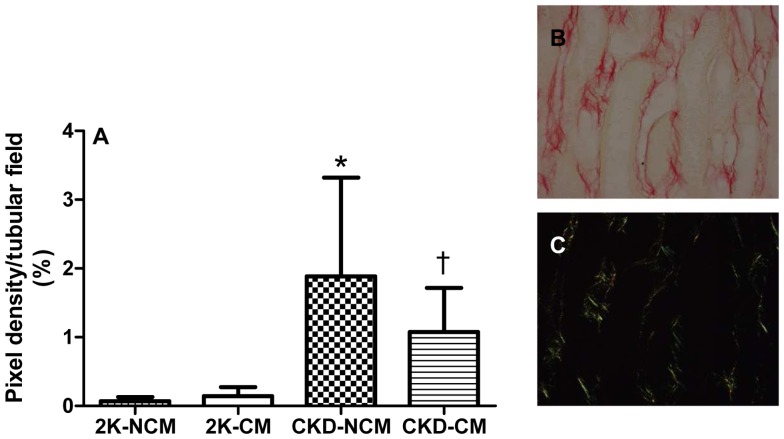
Tubular collagen I and III deposition was reduced after CM treatment in CKD rats. CM treatment reduced sirius red staining (A). Raw (B) and polarized (C) pictures are shown. 2K-NCM (n = 6); 2K-CM (n = 6); CKD-NCM (n = 13); CKD-CM (n = 13). *P<0.05: CKD vs. 2K, †P<0.05: CKD-CM vs. CKD-NCM.

Mean±SD. *P<0.05: 2K vs. CKD;

Compared to healthy rats, both CKD-NCM and CKD-CM-treated rats demonstrated enhanced tubulo-interstitial damage. However, tubular atrophy and interstitial fibrosis were significantly lower after CM treatment in CKD rats (figure 6). Tubular deposition of collagen I and III was decreased in CKD-CM rats compared to CKD-NCM rats (figure 7).

The number of endothelial cells per tubular field was not different between CKD-NCM and CKD-CM treatment, neither were the numbers of tubular ED-1^+^ and CD3^+^ cells. The number of apoptotic cells was not different in glomeruli and tubulo-interstitium of CM and NCM as determined by TUNEL staining (table 3). Exosome treatment had no effect of renal damage (figure S1).

### Cytokine Profile in Remnant Kidney is Altered after CM Treatment

**Figure 8:**
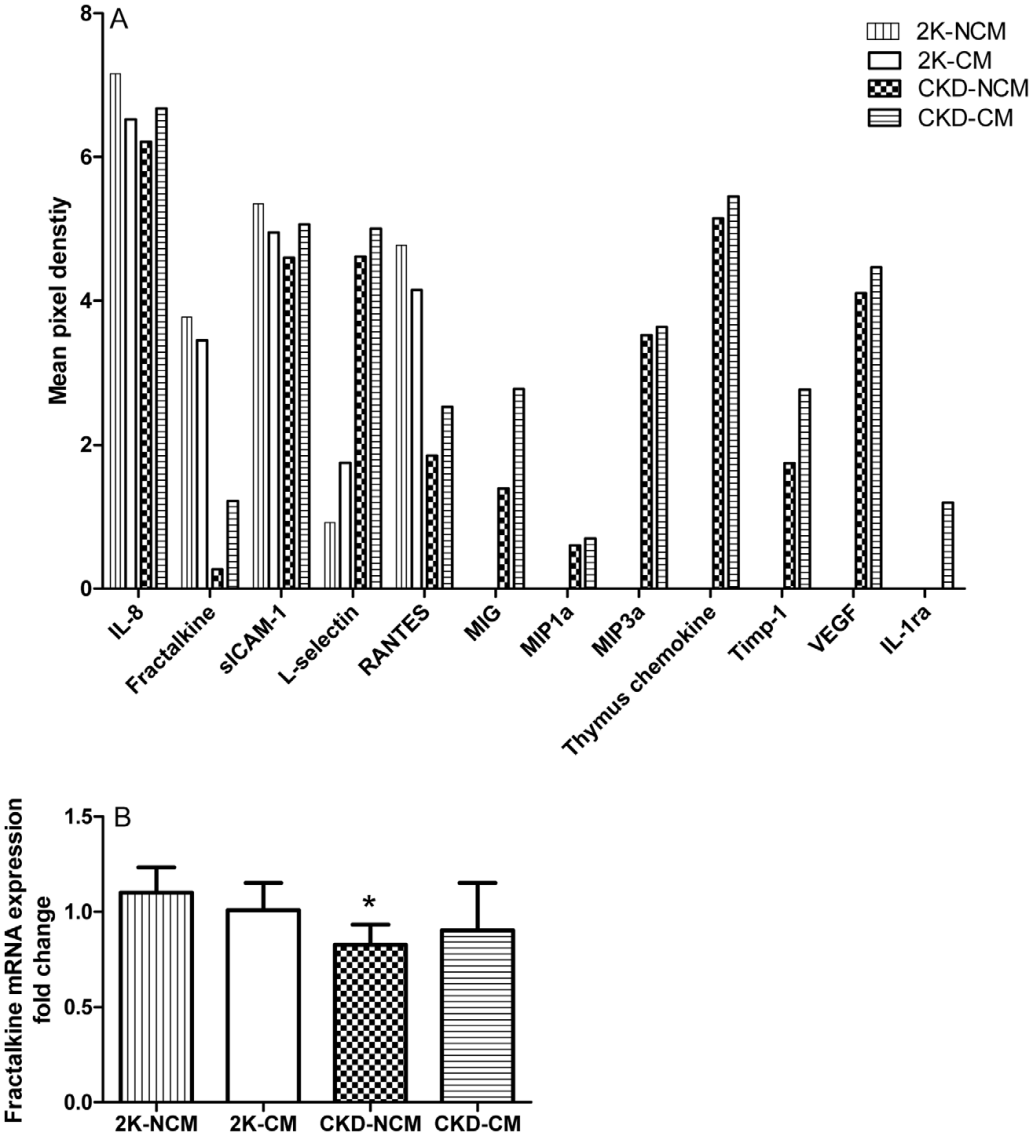
Inflammatory cytokine expression in healthy and CKD kidneys. **A: Cytokine array in array.** Striped bars = 2K-CM (n = 6); dotted bars = 2K-NCM (n = 6); black bars = CKD-NCM (n = 7); white bars = CKD-CM (n = 4). IL-8 = interleukin 8; sICAM = soluble Inter-Cellular Adhesion Molecule; L-selectin = leucocyte cell-adhesion molecule; RANTES = Regulated upon Activation, Normal T-cell Expressed, and Secreted; MIG = Monokine Induced by Gamma-Interferon; MIP1α = Macrophage Inflammatory Protein-1 alpha; MIP3α = Macrophage Inflammatory Protein-3 alpha; Timp-1 = Tissue Inhibitor of Metalloproteinase 1; VEGF = Vascular Endothelial Growth Factor; IL-1RA = Interleukin 1 Receptor Antagonist. **B: Fractalkine mRNA in healthy and CKD kidneys.** Striped bars = 2K-CM (n = 6); dotted bars = 2K-NCM (n = 6); black bars = CKD-NCM (n = 7); white bars = CKD-CM (n = 4). *P<0.05: CKD vs. 2K.

To screen whether CM affected local production of inflammatory cytokines by kidney cells in healthy and CKD kidneys, a cytokine array was performed on kidney homogenates (figure 8a). Seven inflammatory cytokines, Monokine Induced by Gamma-Interferon (MIG); Macrophage Inflammatory Protein-1 alpha (MIP1α); Macrophage Inflammatory Protein-3 alpha (MIP3α); thymus chemokine, Tissue Inhibitor of Metalloproteinase-1 (TIMP-1); VEGF and Interleukin 1 Receptor Antagonist (IL1-RA) were only detected in CKD and not in healthy kidneys.

Fractalkine and Regulated upon Activation, Normal T-cell Expressed, and Secreted (RANTES) were lower in CKD kidneys, whereas L-selectin was higher in CKD kidneys compared to healthy kidneys. In healthy kidneys CM had no significant effects on cytokines, whereas in CKD kidneys CM increased the expression of both Fractalkine and IL-1Ra. Gene expression of Fractalkine was decreased in CKD compared to healthy controls and increased by CM in CKD (figure 8b).

### CM Effectively Induces Angiogenesis and Wound Closure

**Figure 9:**
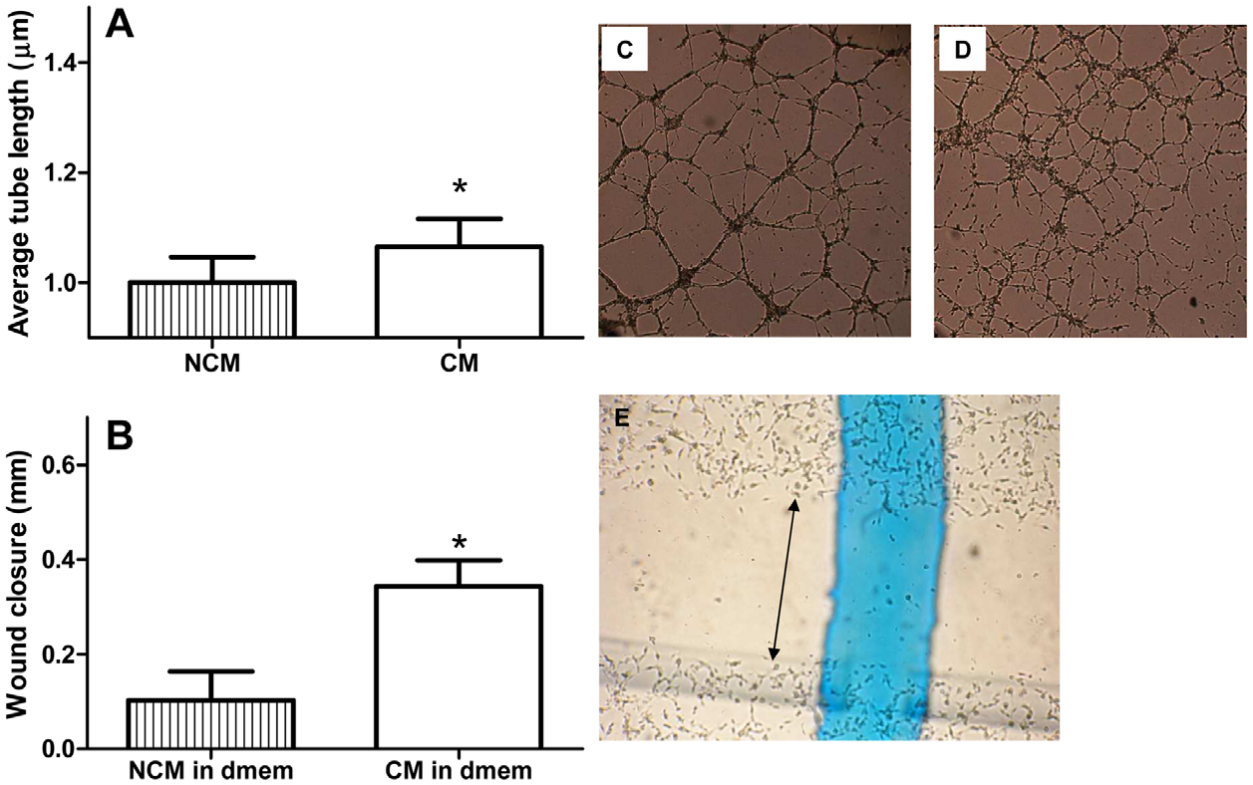
CM stimulates in vitro angiogenesis (A) and wound closure (B). Average tube length was increased after CM treatment compared to NCM (C+D). In a scratch wound assay, CM treatment increased wound (indicated by black arrow in E) closure compared to NCM treatment. *P<0.05 CM vs. NCM.

To evaluate the effectiveness of CM *in vitro*, an angiogenesis assay and wound closure assay were performed. CM was able to significantly induce angiogenesis compared to NCM (figure 9a). Exosome treatment showed also increased angiogenesis (figure S2). We also analysed the effect of CM on wound closure in an *in vitro* scratch wound assay. As PBS induces cell death in the scratch wound assay, PBS could not be used as vehicle or control. We therefore used CM and NCM diluted in DMEM. CM-treated HMECs showed increased wound closure compared to NCM treatment (figure 9b).

## Discussion

Our study demonstrates for the first time that repeated IV administration of human embryonic MSC derived CM as a ‘rescue intervention’– i.e. 6 weeks after CKD induction - can markedly attenuate the reduction of both GFR and ERPF as assessed by classical “gold standard” inulin and PAH clearance methodology and that this effect is detectable at 6 weeks after administration indicating long-term protection. Furthermore, histology showed a reduction in renal injury.

Previous studies showed beneficial effects of MSC administration in models of AKI (reviewed in [5], [7]) and have even led to phase I clinical trials on allogenic MSC administration in AKI (clinicaltrial.gov Identifier: NCT00733876 and NCT01275612). However, the relevance of these observations in AKI to CKD is unclear and data on MSC administration in CKD are sparse. In a few studies, administration of MSC was shown to prevent development of CKD if administered directly after induction of the disease [6], [25]–[28] or at early stages of CKD [29]. However, studies aiming at therapeutic ‘rescue’ in established CKD have not been reported. Furthermore, although most of the reports on beneficial effects suggest that the therapeutic effect is at least in part mediated by paracrine factors secreted by the cells [30], only two studies have applied MSC-CM as therapeutic strategy, and both were performed in AKI models and report conflicting results [12], [13]. More recently, two studies reported beneficial effects of microvesicles derived from human adult MSC-CM in experimental AKI [18], [19].

We intravenously administered human embryonic MSC-CM that was harvested using a clinically compliant protocol. We used the 5/6^th^ nephrectomy ablation model, a known experimental model of progressive renal disease, associated with systemic and glomerular hypertension, capillary loss, renal inflammation and gradual development of glomerulosclerosis, resembling human CKD [31], [32]. Creatinine clearance tends to underestimate the decline in GFR due to extensive tubular creatinine secretion in rats [33], underlining the importance of the gold standard method to determine renal function by inulin and PAH clearance [34]. We previously showed a marked reduction of GFR and ERPF in this model using classic clearance technology, while this was less apparent from changes in plasma creatinine, plasma urea, or creatinine clearance [35]. Injection of CM IV at 6 weeks after induction of CKD, a time point at which kidney failure is established, resulted in a long-term beneficial effect on GFR and ERPF as well as a marked reduction in SBP of up to 27 mmHg, lasting up to at least six weeks post-injection between groups. Besides these functional effects, glomerulosclerosis was reduced and glomerular DNA damage repair increased. Our findings of a beneficial effect of human embryonic MSC secretions on progression of experimental established CKD may have relevance for treatment of human CKD. Clinical use of cell-free MSC secretions may have important advantages over MSC administration, for example with regard to risks of malignant [10], [11] or non-malignant transdifferentiation [8], [9].

Several mechanisms have been proposed for the beneficial effects of paracrine factors secreted by MSC on the injured kidney: immunosuppressive and inflammatory actions, proangiogenic, antifibrotic and anti-apoptotic effects. Our data points at enhanced glomerular endothelial regeneration and genome integrity preservation through active DNA damage signaling. Such enhancement of glomerular endothelial repair has previously been shown to provide protection against glomerulosclerosis progression [36]. We observed a favorable shift in glomerulosclerosis after CM as compared to NCM treatment, which was associated with higher glomerular endothelial cell numbers after CM, whereas glomerular size was not different. Approximately 10% fewer glomeruli were totally sclerotic hence non-functional in CKD-CM rats which match the increase of GFR compared to CKD-NCM rats. The constant filtration fraction points at increased preglomerular and intraglomerular vascular capacity, possibly by preserved endothelial function.

Furthermore, we show that human embryonic MSC CM enhanced endothelial cell migration and angiogenesis *in vitro*. These observations are in line with a report by Togel et al demonstrating *in vitro* vasculotropic effects of adult rat MSC CM [15]. Recently, our human embryonic MSC-CM was also shown to increase capillary density and improve cardiac function after acute myocardial infarction in a pig model [22]. In our CKD model, at 6 weeks after injection of MSC-CM or MSC-NCM we found no differences in the numbers of apoptotic and proliferating cells, nor in the presence of VEGF in the kidney, however, we cannot exclude that anti-apoptotic, mitogenic or VEGF effects have occurred in an earlier stage. We did observe a reduction in tubular inflammation and fibrosis as well as increased expression of fractalkine and Il-1RA, two cytokines that are involved in recruitment of inflammatory response [37], [38] after CM as compared to NCM treatment, suggesting a role for paracrine anti-inflammatory and anti-fibrotic effects, consistent with findings in other disease models [39], [40].

Recent studies on the effects of MSC-derived microvesicles in acute kidney injury models support a potential exosome-mediated renoprotective effect. Bruno et al. reported that human adult MSC-derived microvesicles, which include exosomes, mimicked the protection against AKI as provided by intravenously administered MSC [18]. Gatti et al showed that single administration of human MSC-derived microvesicles immediately after ischemia-reperfusion injury protected against the development of both acute and chronic kidney injury [19]. Furthermore, in a mouse myocardial infarction model it was recently shown that cardioprotection by human embryonic MSC was mediated by exosomes [21]. Based on the above reports we proposed exosomes to be the CM components that provide protection against CKD progression. However, repeated IV administration of human embryonic MSC derived exosomes in our model of established CKD did not affect progression of CKD (text S1).

We used exosome concentrations in CKD rats that were approximately fourfold the concentration of exosomes present in CM, similar to the exosome concentrations previously shown to improve cardiac function after myocardial infarction [21]. Moreover, *in vitro* both human embryonic MSC derived exosomes and CM effectively induced wound closure and angiogenesis. Lack of a significant therapeutic effect of exosomes in this model of chronic renal injury may be due to tissue specific requirements regarding exosome content and/or dose. Our results suggest that the beneficial effect in our model of CKD was mediated by soluble factors and cytokines. Whether rat exosomes would be more effective in the damaged kidney is unknown.

In line with an effect via soluble factors and cytokine, Togel et al. demonstrated that adult rat MSC-CM contains VEGF, HGF and IGF [15] which mediate renoprotection. Previous analysis on the secretory product of human embryonic MSC showed the presence of several gene products that play a role in angiogenesis; kinase insert domain receptor, VEGF, interleukin 8, angiopoietin and fibroblast growth factor [41]. Studies showing that MSC with knockdown of IGF-1 or VEGF failed to protect rats from AKI [14], [16] support a role for proangiogenic factors. Semedo et al. found higher levels of anti-inflammatory cytokines in kidney extracts of MSC-treated animals after ischemia reperfusion injury [29], which is consistent with our observations 6 weeks after CM administration in CKD.

A limitation of our study is that we did not administer CM depleted of exosomes in our CKD model. Therefore we cannot exclude that exosomes mediate the beneficial effect in CKD but need an immunomodulatory or anti-inflammatory factor that is present in the CM. In this respect a recent study may be of interest which reported that porcine MSC have limited immune-modulating activity which abolishes their protective efficacy in AKI [42].

In conclusion, our study demonstrates a marked renoprotective effect of human embryonic MSC derived CM in a rat model with established CKD, as shown by a higher GFR and considerably less glomerular damage after a CM administration. This is probably due to increased endothelial cell regeneration through active DNA damage repair, proliferation and angiogenesis. These findings provide a basis for further research towards potential clinical application of CM-based therapies in human CKD.

## Materials and Methods

### Animals

#### Ethics Statement

The protocol was approved by the Utrecht University committee of Animal Experiments (DEC nr 2007.II.050.131).

#### Animal model

Male inbred Lewis rats (Charles River, Sulzfeld, Germany) were housed under standard conditions in a light-, temperature- and humidity-controlled environment.

CKD was induced in 8-week-old inbred male Lewis rats by two-stage subtotal nephrectomy (SNX) as described (t = 0) [43]. Briefly, the right kidney was removed (wk –1) and one week later (wk 0) the poles of the left kidney were cut off, equalling approximately 66+/−4% of the weight of the previously removed kidney. Progression of CKD was accelerated with L-N^G^-Nitroarginine (L-NNA), a nitric oxide (NO)-synthase inhibitor (20 mg/L) in drinking water for 8 wk, (wk −4 to wk 4), and after wk 0, animals were fed standard powdered chow (CRM-FG; Special Diet Services Ltd., Witham, Essex, UK) supplemented with 6% NaCl.

### MSC-CM Preparation

The protocols for MSC generation and CM preparation have been described previously [41]. In short, a chemically defined serum free culture medium (Dulbecco’s modified eagle medium (DMEM), supplemented with insulin, transferrin, and selenoprotein, fibroblast growth factor 2, platelet derived growth factor AB, glutamine-penicillin-streptomycin, and ß-mercapto-ethanol) was conditioned by MSCs derived from human embryonic stem cells (hESCs) using a clinically compliant protocol. Three polyclonal, karyotypically stable and phenotypically MSC-like cultures that did not express pluripotency-associated markers but displayed MSC-like surface antigens (CD29^+^, CD44^+^, CD49a^+^/e^+^, CD105^+^, CD166^+^, CD34^−^, CD45^−^) and gene expression profile, were generated by trypsinization and propagation of hESCs from either HuES9 hESC line or H1 hESC line in feeder- and serum-free selection media [44]. One of these cultures, HuES9.E1 could be stably expanded for at least 80 population doublings. To harvest MSC secretions, hESC-derived MSC cultures were transferred to a chemically defined, serum free culture medium to condition the medium for three days before the media containing MSC secretions were collected, clarified by centrifugation, concentrated 25 times using 10 kDa MW cut-off ultra-filtration membranes and sterilized by filtration through a 220 nm filter. After these steps, the protein concentration was 0.50 mg/ml. As a negative control, the above-mentioned serum free culture medium was processed equally (non-conditioned medium, NCM). To study the effect of MSC-specific proteins, CM and NCM were diluted in sterile PBS in parallel before administration to reach a protein concentration of 50 µg/250 µl. For MSC-derived exosomes preparation, see text S1.

### Effects of Administration of MSC-derived Conditioned Medium in CKD Rats

At week 5 rats with confirmed CKD were stratified based on plasma urea (>9 mmol/L) and systolic blood pressure (SBP) to receive CM or NCM via tail vein injections (twice daily for 4 consecutive days) at week 6 when stable CKD has developed, as follows: **healthy-NCM** (n = 6), healthy rats received 250 µl NCM per injection; **healthy-CM** (n = 6), healthy rats received 250 µl CM per injection; **CKD-NCM** (n = 13), rats with CKD received 250 µl NCM per injection; **CKD-CM** (n = 13), rats with CKD received 250 µl NCM per injection.

**Figure 10:**
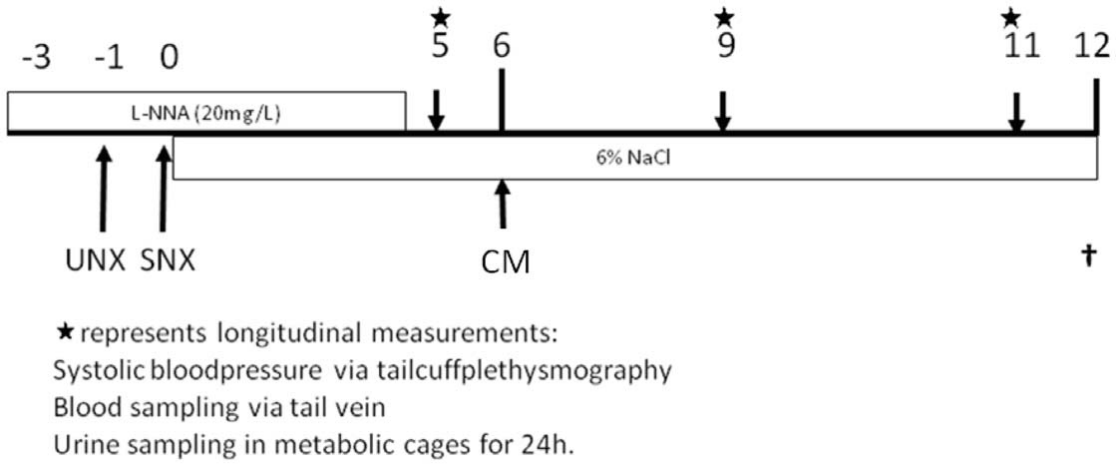
Representation of experimental set up. UNX = uninephrectomy; SNX = subtotal nephrectomy; CM = conditioned medium administration. Stars indicate longitudinal measurements.

At week 12 terminal kidney function was measured under barbiturate anesthesia (see below). Directly thereafter, rats were sacrificed and tissues were collected and either frozen in liquid nitrogen or fixed in 4% paraformaldehyde (PFA) for embedding in paraffin. For detailed time-line, see figure 10. MSC-derived exosomes were studied in the same experimental set-up (text S1).

### Longitudinal Chronic Kidney Disease Evaluation

Rats were weighed weekly. In week 5, 9 and 11, 24 h urine, blood samples were collected and SBP was measured by tail cuff sphygmomanometry [45]. To collect 24 h urine, rats were placed in metabolism cages without food for 24 h, but with free access to water with 2% glucose. Urine was collected on antibiotic/antimycotic solution (Sigma, St. Louis, MO; A5955) and stored at –80°C. Blood samples were collected from the tail vein. Urine protein was measured with Coomassie blue. Sodium and potassium were determined by flame photometry.

### Terminal Kidney Function

Kidney function was assessed by inulin clearance to determine glomerular filtration rate (GFR) and para-ammino hippuric acid (PAH) clearance to determine the effective renal plasma flow (ERPF) as described [45]. Briefly, rats were anesthetized with intraperitoneal pentobarbital sodium (60 mg/kg) and placed on a servo-controlled surgical table that maintained body temperature at 37°C. The trachea was cannulated with a PE-10 cathether. A PE-50 catheter was placed in the left jugular vein for infusion of solutions and a PE-10 catheter was introduced in this PE-50 cathether for supplemental anesthetic. The left femoral artery was cannulated with PE-50 tubing for measurement of mean arterial pressure (MAP) and blood sampling. A PE-50 catheter was placed in the bladder for urine collection. During surgery, animals received an intravenous infusion of a 150 mM NaCl solution containing 6% bovine serum albumin (BSA). Following surgery, the infusion was switched to a 150 mM NaCl solution with 1% BSA at the same infusion rate. This infusion was maintained throughout the experiment. The solution also contained inulin and para-amino hippurate (PAH) for clearance measurements. A 60-min equilibration period was observed before the start of the 60-min clearance measurements. During this clearance measurement urine was sampled for 15 minute periods and before and after the clearance measurement blood was sampled. Clearances and fractional excretions were calculated by standard formulae. Renal blood flow was calculated from ERPF and hematocrit.

### Renal Morphology

Glomerulosclerosis and tubular interstitial damage were scored on 3 µm periodic acid Schiff (PAS)-stained paraffin-embedded slides [46]. Collagen I and III contents was stained with Sirius red, visualized with circular polarized light and digitally analyzed using ImageJ software [46]. The percentage of collagen area was calculated by dividing the Sirius red stained area by the total image area. Monocytes/macrophages (ED-1 stain) and leucocytes (CD3 stain) were counted in glomeruli and tubulo-interstitum [47]. Terminal deoxynucleotidyl transferase-mediated dUTP-biotin nick end labeling (TUNEL) staining (Apoptag Plus in situ Peroxidase kit, Millipore, Temecula, CA, USA) was performed according to manufacturer guidelines. The number of apoptotic cells was determined as the number of TUNEL-positive cells in the images of 50 randomly selected fields (x200 magnification) per section. Endothelial cells were stained with JG12 (Bender Medsystems GmbH, Vienne, Austria) after heat antigen retrieval in citrate buffer (pH 6.0) [48]. JG12 positive cells were determined in the glomeruli (calculated in at least 100 glomeruli per animal) and in peritubular areas (calculated in 20 peritubular fields per animals) using Adobe Photoshop software, version 8.0.1 (Adobe Systems; San Jose, CA) and ImageJ software, version 1.42q (National Institutes of Health; Bethesda, MD). To score nuclei repairing DNA damage, paraffin-embedded kidney sections were deparafinized and treated with PO block for 15 minutes and incubated at 100°C in Citrate/HCL buffer for 20 minutes. The sections were stained with mouse anti-γH2AX (ser139) (Millipore, 1∶200) overnight at 4°C. Polyclonal rabbit anti-mouse HRP (Dako, 1∶100) was incubated 30 minutes at RT. Finally, BrightVision Poly HRP-Anti Rabbit IgG (Immunologic) was incubated for 1 hour RT. Nova RED substrate kit for Peroxidase (Vector, SK-4800) was used and counterstained with hematoxyline. Analysis was performed using the Aperio ImageScope software.

### Cytokine Array and Gene Expression in Renal Tissue

Kidney samples were collected at termination (6 weeks after CM or NCM administration) and quickly frozen in liquid nitrogen. A rat cytokine array (R&D systems) was performed on kidney homogenates according to manufacturer’s instructions to screen whether CM treatment stimulated local secretion of specific inflammatory cytokines by the host kidney cells. Samples were pooled per treatment group and equal amounts of protein were loaded on the blots. From all pooled samples blots were performed in duplicate and averages of these two pixel densities were used to calculate the average density with Image J software. Background staining and spot size were analysed as recommended by the manufacturer. Briefly, pictures were converted to 8-bit inverted jpeg files and spots were encircled. Per blot, equal spot sizes were analysed.

To determine whether a local production of cytokines could be confirmed on mRNA level, cDNA was isolated from frozen remnant kidney tissue and expression of fractalkine was determined using quantitative real-time RT-PCR (ABi PRiSM 790Sequence Detection SYStem, applied Biosystems, Foster City, CA). The following TaqMan® Gene Expression Assays (Applied Biosystems) were used: (fractalkine (CX3CL1): Rn00593186_m1), (ß-actin: Rn00667869_m1) and (calnexin: Rn00596877_m1). Reactions were carried out in duplicate. Cycle time (Ct) values for fractalkine were normalized for mean Ct-values of Calnexin and β-actin, which we previously determined to be the two most stable housekeeping genes across all groups using the geNorm-program (http://medgen.ugent.be/~jvdesomp/genorm/), and expressed relative to a calibrator (the sample with the lowest expression: the 2K controls), using the ΔΔCt-method. Hence, steady state mRNA levels were expressed as *n*-fold difference relative to the calibrator.

### In vitro Angiogenesis Assay

The potential of CM to stimulate angiogenic tube formation was assessed *in vitro*. For this, 10 µl matrigel (Millipore, Temecula, CA, USA) was added in the inner compartment of an ibidi μ-angiogenesis slide (Ibidi, Munchen, Germany). After the matrigel had solidified, 50 µl of tests-suspension was added, containing respectively 10 µg CM or 10 µg NCM. Subsequently, 10 µl unsupplemented MCDB medium containing 10.000 trypsinized human microvascular endothelial cells (HMEC-1) cells (HMECs; Centers for Disease Control and Prevention, Atlanta, USA) was added. The angiogenesis area was photographed using light microscopy after 18 hours incubation at 37°C, 5% CO_2_ and the mean tubule length, used as a measure of angiogenesis, was determined using Angioquant software [49]. Each sample was assayed in triplicate. The angiogenic potential of MSC-derived exomes was also studied (see text S1).

### In vitro Scratch Wound Assay

The potential of CM to stimulate endothelial cell migration was assessed by *in vitro* scratch wound assay. A mechanical scratch was created with a pipet tip in a confluent monolayer of HMECs. After washing with PBS, 200 µl DMEM medium containing respectively 40 µg CM or 40 µg NCM was placed on the cells. DMEM without supplementation served as control. Reference lines were made on the bottom of the wells to obtain exactly the same field during image acquisition. The scratched area was photographed using light microscopy at start and after 6 hours incubation (37°). The extent of closure after 6 hours was determined relative to the starting width of the scratch (Image-Pro plus software, Media Cybernetics 3.0). Each sample was measured in two wells and two picture-fields per well were examined. Results were averaged for analysis.

### Statistical Analyses

Data are presented as mean ± standard deviation and analyzed by analysis of variance (One-way ANOVA with a Newman-Keuls post-test, Two-way ANOVA with a Newman-Keuls post-test) or Student’s T-test, where appropriate. P<0.05 was considered significant.

## Supporting Information

Figure S1 **Glomerulosclerosis and tubulo-interstitial damage after exosome treatment. A: Segmental (segm) and total (tot) glomerulosclerosis (GS); B:** **Tubulo-interstitial damage.** Exosomes (n = 8); PBS (n = 7). There were no significant differences.
(TIF)

Figure S2 **Exosomes stimulates in vitro angiogenesis.** Average tube length was increased after exosome treatment compared to PBS. *P<0.05: exosomes vs. PBS
(TIF)

Table S1 **Terminal kidney function measurements in the exosome and PBS groups.** There were no significant differences.
(DOCX)

Table S2 **Longitudinal measurements after exosome treatment at week 6 after SNX.** Week numbers indicate the week after SNX. Week 5 represents the week before treatment. There were no significant differences.
(DOCX)

Text S1 **Supporting text including materials and methods, results and references.**
(DOCX)
