# Supplementary figures and images for: Human Embryonic Mesenchymal Stem Cell-Derived Conditioned Medium Rescues Kidney Function in Rats with Established Chronic Kidney Disease
Source: PLoS One. 2012 Jun 19;7(6):e38746. doi: 10.1371/journal.pone.0038746 (PMC3378606; doi:10.1371/journal.pone.0038746)

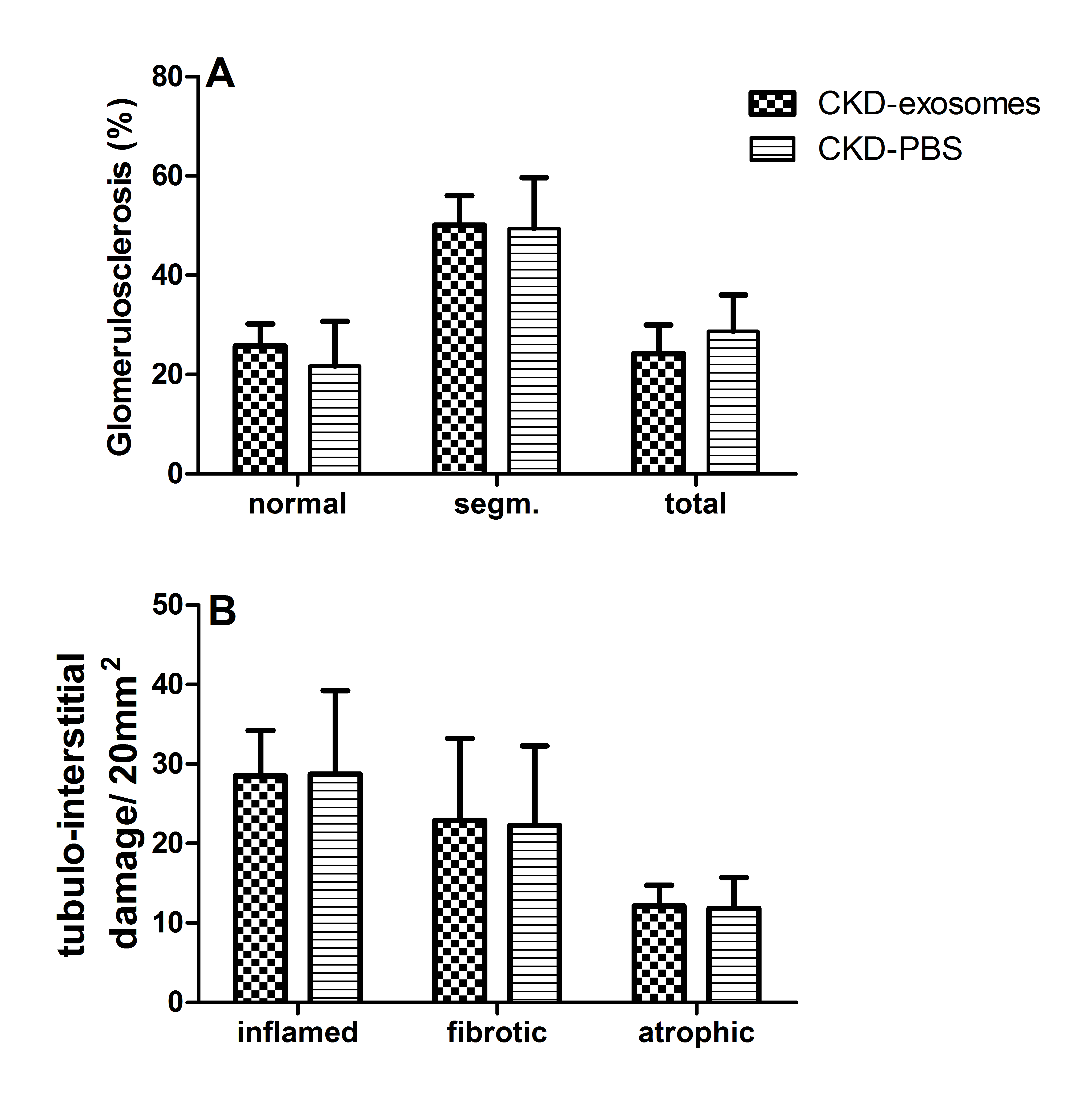

Supplement: Figure S1 — Glomerulosclerosis and tubulo-interstitial damage after exosome treatment. A: Segmental (segm) and total (tot) glomerulosclerosis (GS); B: Tubulo-interstitial damage. Exosomes (n = 8); PBS (n = 7). There were no significant differences. (TIF) [file pone.0038746.s001.tif]

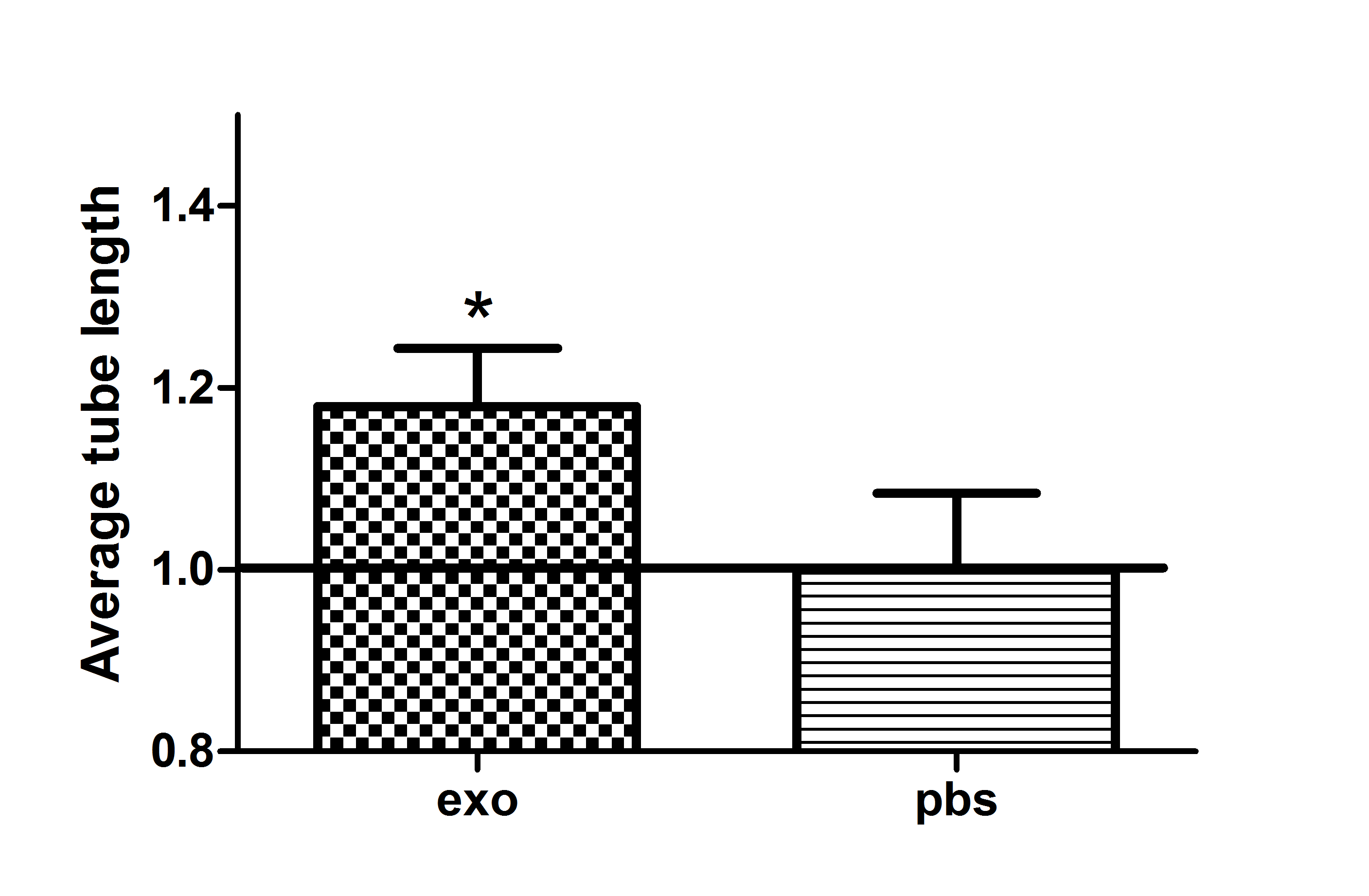

Supplement: Figure S2 — Exosomes stimulates in vitro angiogenesis. Average tube length was increased after exosome treatment compared to PBS. *P<0.05: exosomes vs. PBS (TIF) [file pone.0038746.s002.tif]
