# Supplementary material for: Human Embryonic Mesenchymal Stem Cell-Derived Conditioned Medium Rescues Kidney Function in Rats with Established Chronic Kidney Disease
Source: PLoS One. 2012 Jun 19;7(6):e38746. doi: 10.1371/journal.pone.0038746 (PMC3378606; doi:10.1371/journal.pone.0038746)
Supplement: Table S1 — Terminal kidney function measurements in the exosome and PBS groups. There were no significant differences. (DOCX) [file pone.0038746.s003.docx]

|  | **CKD-exosomes**  n=8 | **CKD-PBS**  n=7 |
| --- | --- | --- |
| MAP (mm HG) | 122±32 | 122±22 |
| GFR (ml/min/100gr) | 0.31±0.13 | 0.32±0.07 |
| ERPF (ml/min/100gr) | 1.12±0.39 | 0.99±0.33 |
| RBF (ml/min/100gr) | 1.93±0.76 | 1.71±0.59 |
| RVR (mmHg/ml/min) | 18±8 | 24±17 |
| Hematocrit | 43±5 | 46±4 |
| FF | 0.28±0.05 | 0.28±0.04 |
| FeNa (%) | 0.96±0.65 | 1.43±1.53 |
| FeK (%) | 44±9 | 38±8 |

**Table S1**

**MAP= mean arterial pressure. GFR=glomerular filtration rate. ERPF-effective renal plasma flow. RBF=renal blood flow. RVR=renal vascular resistance. FF=filtration fraction. FeNa=fractional excretion of sodium. FeK=fractional excretion of potassium**
