# Supplementary material for: Human Embryonic Mesenchymal Stem Cell-Derived Conditioned Medium Rescues Kidney Function in Rats with Established Chronic Kidney Disease
Source: PLoS One. 2012 Jun 19;7(6):e38746. doi: 10.1371/journal.pone.0038746 (PMC3378606; doi:10.1371/journal.pone.0038746)
Supplement: Table S2 — Longitudinal measurements after exosome treatment at week 6 after SNX. Week numbers indicate the week after SNX. Week 5 represents the week before treatment. There were no significant differences. (DOCX) [file pone.0038746.s004.docx]

**Table S2**

|  | **CKD-exosomes**  n=8 | **CKD-PBS**  n=7 |
| --- | --- | --- |
| **Systolic blood pressure (mm Hg)** |  |  |
| wk 5 | 146±15 | 150±10 |
| wk 9 | 158±17 | 160±16 |
| wk 11 | 162±16 | 172±15 |
| **Proteinuria (mg/24h)** |  |  |
| wk 5 | 13±3 | 12±4 |
| wk 9 | 40±28 | 29±18 |
| wk 11 | 44±12 | 39±11 |
| **Urea (mmol/L)** |  |  |
| wk 5 | 10.6±0.9 | 10.4±0.9 |
| wk 9 | 10.7±1.4 | 10.5±0.8 |
| wk 11 | 10.5±1.1 | 9.9±0.5 |
| **Creatinine clearance (ml/min)** |  |  |
| wk 5 | 1.16±0.14 | 1.20±0.16 |
| wk 9 | 1.47±0.33 | 1.43±0.26 |
| wk 11 | 1.19±0.17 | 1.22±0.23 |
