# Supplementary material for: Human Embryonic Mesenchymal Stem Cell-Derived Conditioned Medium Rescues Kidney Function in Rats with Established Chronic Kidney Disease
Source: PLoS One. 2012 Jun 19;7(6):e38746. doi: 10.1371/journal.pone.0038746 (PMC3378606; doi:10.1371/journal.pone.0038746)
Supplement: Text S1 — Supporting text including materials and methods, results and references. (DOCX) [file pone.0038746.s005.docx]

**Supplemental text**

**Materials and methods**

*MSC-CM derived exosome preparation*

The protocols for MSC-CM derived exosome generation and preparation have been described by Lai et al[1]. Exosomes form approximately 3% of the CM by protein weight.

*Effects of administration of MSC-derived exosomes in CKD rats*

To investigate whether exosomes could be the renoprotective factor of MSC-derived CM, the effect of MSC-derived exosomes was studied in the same experimental setting as CM.

The dose of exosomes was extrapolated from a dose that was effective in reducing myocardial injury after coronary ligation in mice [1]. In this experiment at wk 6, rats received MSC-derived exosomes or PBS via tail vein injections, twice daily for 4 consecutive days: **CKD-exosomes** (n=8), rats with CKD received 7 µg exosomes in 250 µl PBS per injection; **CKD-PBS** (n=7), rats with CKD received 250 µl PBS per injection. Longitudinal follow-up and terminal measurements were performed as described before.

*In vitro angiogenesis assay*

The potential of CM and exosomes to stimulate angiogenic tube formation was assessed *in vitro*. For this, 10 μl matrigel (Millipore, Temecula, CA, USA) was added in the inner compartment of an ibidi μ-angiogenesis slide (Ibidi, Munchen, Germany). After the matrigel had solidified, 50 μl of tests-suspension was added, containing respectively 1.4 μg exosomes or PBS. Subsequently, 10 μl unsupplemented MCDB medium containing 10.000 trypsinized human microvascular endothelial cells (HMEC-1) cells (HMECs; Centers for Disease Control and Prevention, Atlanta, USA) was added. The angiogenesis area was photographed using light microscopy after 18 hours incubation at 37°C, 5% CO_2_ and the mean tubule length, used as a measure of angiogenesis, was determined using Angioquant software [2]. Each sample was assayed in triplicate.

**Results**

**Exosome treatment does not reduce CKD progression or renal damage.**

To investigate whether exosomes would be the CM component responsible for the renoprotective effects, exosomes derived from MSC-CM were administered in rats with established CKD and compared with vehicle (PBS). Exosomes did not influence GFR, ERPF, hematocrit, or renal blood flow (supplemental table 1). No differences were observed in MAP, RVR, filtration fraction and fractional excretion of sodium and potassium between exosome- and PBS-treatment (supplemental table 1). SBP, proteinuria, urea, diuresis, creatinine clearance (supplemental table 2), glomerulosclerosis and tubular damage (supplemental figure 1) were also not different between CKD-exosomes or CKD-PBS rats.

**CM and exosomes stimulate tube formation in vitro**

To evaluate the effectiveness of exosomes and CM *in vitro*, an matrigel tube formation assay was performed. Both CM and exosomes were able to significantly induce tube formation compared to their respective controls, i.e. NCM and PBS (supplemental figure 2).

Supplemental Reference List

1. Lai RC, Arslan F, Lee MM, Sze NS, Choo A, et al. (2010) Exosome secreted by MSC reduces myocardial ischemia/reperfusion injury. Stem Cell Res 4: 214-222.

2. Niemisto A, Dunmire V, Yli-Harja O, Zhang W, Shmulevich I (2005) Robust quantification of in vitro angiogenesis through image analysis. IEEE Trans Med Imaging 24: 549-553.
